# Conservative Treatment of Stage IA1 Cervical Carcinoma Without Lymphovascular Space Invasion: A 20-year Retrospective Study in Brazil

**DOI:** 10.1055/s-0043-1769000

**Published:** 2023-05-24

**Authors:** Daniele Lima Alberton, Mila Pontremoli Salcedo, Raquel Potrich Zen, Charles Francisco Ferreira, Kathleen Schmeler, Suzana Arenhart Pessini

**Affiliations:** 1 Obstetrics and Gynecology Department, Irmandade Santa Casa de Misericórdia de Porto Alegre, Porto Alegre, Brazil; 2 Obstetrics and Gynecology Department, Hospital de Clínicas de Porto Alegre, Porto Alegre, Brazil; 3 Department of Gynaecologic Oncology & Reproductive Medicine, The University of Texas MD Anderson Cancer Center, Houston, Texas, United States; 4 Internal Medicine, Irmandade Santa Casa de Misericórdia de Porto Alegre, Porto Alegre, Brazil; 5 Universidade Federal do Rio Grande do Sul, Porto Alegre, Brazil

**Keywords:** Uterine cervical neoplasms, Conization, Conservative treatment, Recurrence, Squamous cell carcinoma, Neoplasias do colo do útero, Conização, Tratamento conservador, Recorrência, Carcinoma de células escamosas

## Abstract

**Purpose:** To evaluate recurrence rates and risk factors among women with stage IA1 cervical cancer without lymph vascular space invasion managed conservatively.

**Methods:** retrospective review of women with stage IA1 squamous cervical cancer who underwent cold knife cone or loop electrosurgical excision procedure, between 1994 and 2015, at a gynecologic oncology center in Southern Brazil. Age at diagnosis, pre-conization findings, conization method, margin status, residual disease, recurrence and survival rates were collected and analyzed.

**Results:** 26 women diagnosed with stage IA1 squamous cervical cancer without lymphovascular space invasion underwent conservative management and had at least 12 months follow-up. The mean follow-up was 44.6 months. The mean age at diagnosis was 40.9 years. Median first intercourse occurred at age 16 years, 11.5% were nulliparous and 30.8% were current or past tobacco smokers. There was one Human immunodeficiency virus positive patient diagnosed with cervical intraepithelial neoplasia grade 2 at 30 months after surgery. However, there were no patients diagnosed with recurrent invasive cervical cancer and there were no deaths due to cervical cancer or other causes in the cohort.

**Conclusion:** Excellent outcomes were noted in women with stage IA1 cervical cancer without lymphovascular space invasion and with negative margins who were managed conservatively, even in a developing country.

## Resumo

**Objetivo:** Avaliar recidiva e seus fatores de risco em mulheres com câncer do colo do útero estádio IA1 sem invasão do espaço linfovascular tratadas conservadoramente.

**Métodos:** Estudo de coorte retrospectivo de pacientes com câncer do colo do útero IA1 escamoso submetidas a cone do colo do útero, entre 1994 e 2015, em um centro de ginecologia oncológica do sul do Brasil. Foram revisados e analisados idade no diagnóstico, achados pré-conização, método de conização, margens, doença residual, recorrência e sobrevida.

**Resultados:** 26 mulheres diagnosticadas com câncer do colo do útero estádio escamoso sem invasão do espaço linfovascular foram submetidas a tratamento conservador, com seguimento mínimo de 12 meses. O tempo médio de seguimento foi 44,6 meses. A média de idade no diagnóstico foi 40,9 anos. A primeira relação sexual ocorreu aos 16 anos (mediana), 11,5% eram nulíparas e 30,8% eram tabagistas atuais ou passadas. Houve um caso de recidiva de neoplasia intraepitelial cervical grau 2 aos 30 meses em uma paciente com vírus da imunodeficiência humana. Não houve pacientes diagnosticados com câncer de colo do útero invasor recorrente, e não houve mortes por câncer do colo do útero ou outras causas.

**Conclusão:** Os resultados observados após tratamento conservador em mulheres com câncer cervical escamoso IA1 sem invasão do espaço linfovascular com margens negativas foram excelentes, mesmo em um país em desenvolvimento.

## Introduction

Cervical cancer (CC) is the fourth cancer in global incidence and the fourth leading cause of death due to cancer in women. Greater than 85% of cervical cancer deaths occur in low- and middle-income countries (LMICs) where it is the first or second leading cause of cancer-related deaths. 1 CC is the third most common cancer and the fourth most common cause of cancer-related death among women in Brazil. 2 The highest incidence occurs among women in reproductive ages, which reinforces the need for safe oncologic outcomes as well as fertility-sparing approaches. 2

Micro-invasive cervical cancer was initially described in 1947 by Mestwerdt 3 and extensively revised. According to the 2018 *International Federation of Gynecology and Obstetrics* (FIGO) new definition, stage IA disease is defined as invasive carcinoma diagnosed only by microscopy with a depth of invasion up to 5mm. 4 5 The horizontal dimension (up to 7mm extension) is no longer considered. Stage IA1 disease has stromal invasion up to 3mm, and stage IA2 has stromal invasion greater than 3mm and up to 5mm. Lymphovascular space invasion (LVSI) does not modify the stage, but it may impact prognosis and therapeutic approach. 4 6

FIGO proposes extrafascial hysterectomy or conization with negative margins as treatment options for stage IA1 CC without LVSI, but the studies that evaluated stage IA1 treatment options have a lack of homogeneity regarding variables such as LVSI, depth of invasion, histologic type, and surgical margin status. 4 7 8 9 10 11 12 13 14 15 16 17 In addition, risk factors for cervical cancer have been extensively studied, but not in this specific population. 18

There are limited reports from LMIC on cervical micro-invasive cancer. The purpose of this study was to analyze recurrence rates after conservative treatment for stage IA1 squamous cell CC without LVSI and with negative cone margins. In addition, we sought to describe the population characteristics.

## Methods

A retrospective cohort study of patients diagnosed with stage IA1 squamous cell CC without LVSI who underwent loop electrosurgical excision procedure (LEEP) or cold knife cone biopsy (CKC), between June 1994 and December 2015, at the Gynecology of the Federal University of Health Science of Porto Alegre (UFCSPA)/Hospital Irmandade Santa Casa de Misericórdia de Porto Alegre (ISCMPA), was performed after Ethics and Research Committee ISCMPA approval (No. 2.606.2990, Irmandade Santa Casa de Misericórdia de Porto Alegre, Porto Alegre, Brazil). All methods were performed in accordance with the relevant guidelines and regulations as well as in compliance with the requirements of the Declaration of Helsinki. The need for informed consent was waived by Ethics Committee ISCMPA, due to the retrospective nature of the study.

The study population included stromal invasion up to 3mm, with surface extension no greater than 7mm (previous FIGO staging guidelines). Exclusion criteria included positive surgical margins, non-conservative surgery, and less than 12 months of follow-up. The following variables were analyzed: age at diagnosis, age at first intercourse, parity, comorbidities related to the immunosuppressive status, tobacco use, contraceptive method, menopausal status, pre-conization cytology results, biopsy pathologic results, conization method, conization pathologic results, margin status, residual disease, follow-up, and recurrence. A negative margin was defined as the absence of cervical intraepithelial neoplasia or carcinoma at the surgical margins of the CKC/LEEP specimen. Risk factors for CC were defined as age at first intercourse below 15 years, parity, immunosuppression, current or past smoking, and use of oral contraceptives. 19 20 21 22 23

All the surgical procedures were performed by UFCSPA/ISCMPA gynecologic oncology team, and experienced pathologists analyzed the samples. Follow-up included pelvic examination with cytology and colposcopy for the following 5 years after the first treatment. Patients were evaluated every 3 months for the first 2 years and, every 6 months thereafter. The presence of a high-grade squamous intraepithelial lesion (HSIL)/ cervical intraepithelial neoplasia (CIN) 2/3 or carcinoma after 6 months post-treatment defined recurrence; before 6 months, findings were considered disease persistence.

Continuous variables were expressed as mean and standard error of mean (± SEM), or by median and 95% Confidence Interval [95%CI]. Categorical variables were described as absolute (n) and relative (n%) frequencies. The Shapiro-Wilk test was used to determine the normality of data distribution. Spearman's correlations were carried out among all variables. Statistical analysis was performed using SPSS, version 18.0. [SPSS Inc. Released 2009. PASW Statistics for Windows, Version 18.0. Chicago: SPSS Inc.].

## Results

A total of 50 patients had the diagnosis of stage IA1 CC without LVSI. Twenty-six patients underwent conservative treatment with CKC or LEEP and were included in the study. Demographic and clinical characteristics are shown in Table 1 .

**Table 1:** Demographic and clinical characteristics

Characteristic	Total (n = 26)
Age (years) (mean ± SEM)	40.9 ± 2
Parity (mean ± SEM)	2.8 ± 0.3
Age of first Intercourse (years) (median [CI95%] ^a^ )	16 [15.2–17.3]
Parity – n (n%) 0 1-4 ≥ 5	3 (11.5) 20 (76.9) 3 (11.5)
HIV positive serology n (n%) Yes No	1 (3.8) 25 (96.2)
Tobacco use n (n%) Current smoker Former smoker Non-smoker	5 (19.2) 3 (11.5) 18 (69.2)
Contraceptive method – n (n%) Oral contraceptive pills Barrier Tubal ligation None	12 (46.2) 2 (7.7) 2 (7.7) 10 (38.5)

The mean age at diagnosis was 40.9 years (23 to 59), 4/26 (15.4%) participants were younger than 30 years, 4/26 (15.4%) were 50 years or older, and 8/26 (30.8%) were menopausal status. The median age at first intercourse was 16 (range 15-17) years. The Mmean parity was 2.8 ± 0.3; 3/26 (11.5%) were nulliparous and 3/26 (11.5%) had five or more children. One patient (3.8%) was Human immunodeficiency virus (HIV)-positive. Previous or current use of tobacco was reported in 8/26 (30.8%) women. The contraceptive method used by 12/26 patients (46.2%) was oral contraceptive pills (p < 0.01). Only two women (7.7%) did not have at least one of the CC risk factors. Among 15/26 (57.7%) had one risk factor, 5/26 (19.2%) had two risk factors and 4/26 (15.4%) had three risk factors. Pre-conization cytology was low-grade squamous intraepithelial lesion (LSIL) in 2/26 (7.7%), atypical squamous cells of undetermined significance (ASC-US) in 1/26 (3.8%), HSIL in 18/26 (69.2%), atypical squamous cells cannot exclude a higher-grade lesion (ASC-H) in 2/26 (7.7%) and invasive carcinoma in 1/26 cases (3.8%). In the other 2/26 cases (7.7%) cytology was negative. Cervical biopsies prior to cone showed CIN 1 in 2/26 (7.7%), CIN 2 or CIN 3 in 14 of 26 (53.8%) cases, and micro-invasive carcinoma was identified in six of 26 (23.1%) women. In the remaining four out of 26 cases, (15.4%) a cervical biopsy was negative or not performed, and diagnostic conization was recommended ( Table 2 ).

**Table 2:** Pap Test and pathology report previous conization

Variable	Total (n = 26)
Pre-surgery Pap test – n (n%) LSIL HSIL ASC-US ASC-H Invasive carcinoma Negative results	2 (7.7%) 18 (69.2%) 1 (3.8%) 2 (7.7%) 1 (3.8%) 2 (7.7%)
Pre-surgery cervical biopsy – n (n%) CIN 1 CIN 2/3 Invasive carcinoma Negative results or not performed	2 (7.7%) 14 (53.8%) 6 (23.1%) 4 (15.4%)

The surgical treatment was CKC in 23 out of 26 (88.5%) and LEEP in three of 26 (11.5%) patients. The depth of the CKC pieces ranged from 0.7 to 4.8 centimeters. The three LEEP procedure comprised type 1 and 2 excisions. The cone results were CIN 1 in 1/26 cases (3.8%), CIN 2/3 in 2/26 (7.7%), and micro-invasive carcinoma in 23/26 (88.5%). Three of 26 cases (11.5%) were diagnosed with micro-invasive carcinoma only in the cervical biopsy performed prior to the cone ( Table 3 ). Stromal invasion depth up to 1mm was found in 11 of 26 patients (42.3%). As soon as the cone biopsy was performed, 5/26 (19.2%) required a repeat intervention due to positive endocervical margins with CIN 3. In the remaining 21/26 (80.8%) patients, the surgical margins were negative. Only two of the five cases with positive margins (40%) showed residual disease with CIN 3, and none with invasion (an = 5 in Table 3 ). All five women that underwent a second cone had negative margins in the second procedure.

**Table 3:** Surgical and pathologic findings

Surgical and Pathological findings	Total (n = 26)
Technique – n (n%) CKC LEEP	23 (88.5) 3 (11.5)
Conization anatomopathological results cone – n (n%) CIN 1 CIN 2/3 Micro-invasion	1 (3.8) 2 (7.7) 23 (88.5)
Cone biopsy negative margins (CIN 3) – n (n%) Yes No	21 (80.8) 5 (19.2)
Reconization results – n (n%)* Negative CIN 3	3 (60) 2 (40)
Follow-up period (months) – mean ± SEM	44.6 ± 4.6

*n=5 .

One patient recurred at 30 months with CIN 2 in an HIV-positive woman, and she was retreated with CKC. None of the patients developed recurrent invasive carcinoma and no patients died of cervical cancer. The studied variables did not correlate with recurrence (p > 0.05). The mean follow-up was 44.6 months (12 to 98). Sixteen of 26 (61,5%) women were followed for more than 36 months.

## Discussion

The primary finding from our study confirms that CKC or LEEP are an alternative and effective treatment in conservative management of women with IA1 squamous CC without LVSI and negative margin, even in low- and middle-income countries. This conservative management resulted in only one (3.8%) recurrence of CIN 3 in a 50-year-old woman with HIV.

The main risk factor for the development of CC is the persistent infection of carcinogenic types of Human papillomavirus (HPV), especially the subset of HPV16. 21 It happens in a small percentage of women and becomes more prevalent after 30 years. 21 24 25 26 27 28 29 30 The current study identified the mean age at CC diagnosis at 40 years, similar to other studies. 6 15 16 17 31 As far as age is concerned in stage IA1 CC, 4/26 patients were 50 years or older and it was not correlated to recurrence in this study, but it was associated with a greater chance of recurrence in previous studies. 6 10 32 Elliot and collaborators studied 476 stage IA CC and they found there was a tendency for more recurrences in older women in a univariate analysis. 10 Hartman et al., 32 in a large and recently published study involving 562 women treated by cervical conization or hysterectomy found recurrence twice as frequent in women over 40 years of age.

Another important risk factor for CC is HIV co-infection, which is linked to accelerated progression of pre-cancerous lesions and more frequent recurrences. Immunosuppression is associated with prevalence of HPV infection and viral persistence. However, HIV-infected but well-controlled with high-activity antiretroviral therapy presents a similar evolution to other women. 22 Although in this study the only CIN recurrence was in an HIV patient, this data is not enough to draw conclusions.

Studies have shown that the risk of cervical cancer increases with the increasing time of use of oral contraceptive pills. 20 But the use of oral contraceptive pills, the most common contraceptive method in this population, did not correlate with recurrence. Early age at first intercourse, high parity, and use of tobacco, risk factors for cervical cancer, also did not correlate with cervical cancer recurrence in this study. 21 22 23 33

Previous studies about CC stage IA recurrence rates range between 1.7% to 9.6% and include CIN 2, CIN 3, intraepithelial vaginal neoplasia (VAIN) 3, and invasive cancer. 10 12 14 15 16 17 31 32 The variation in recurrence frequencies may be explained by the heterogeneity in the use of different definitions of recurrence and different methodologies. Hartman et al. 16 described a study performed in Brazil that showed a 7% (3/41 cases) recurrence rate and included one case that recurred with CIN 3/VAIN 3, one case with micro-invasive carcinoma and one with invasive carcinoma IB1 at 6, 9 and 104 months after completion of treatment respectively. Lee et al. 17 described a retrospective study with 22 conservative management in 75 stage IA1 CC patients, performed in three affiliated hospitals in South Korea, that showed two cases, out of the 6 recurrence cases, with micro-invasive carcinoma. The low recurrence rate found in our study can be attributed to the effects of the surgical technique used in which the surgical margins of the remaining cervix were cauterized during hemostasis which may eliminate residual neoplastic cells; this technique was not described by Hartman et al. 16 Xiang et al., 31 described that maybe the cautery used for hemostasis was related to their recurrence reduction. 31 However, their recurrence rate was still higher (9.7%), but they included cases in which very early recurrence happens (3 months after conization) potentially representing persistent disease.

The low recurrence rate in our cohort may be also attributed to the high number (42%) of cases with stromal invasion ≤ 1mm. Costa et al. 34 evaluated 230 CC IA1-2 women primarily conservatively treated and they detected seven recurrences (3%) for invasive lesion closely related to the depth of stromal invasion (0/110 tumors with stromal invasion ≤1mm, 2/63 in tumors with invasion of 1.1 to 3.0 mm and 5/57 among tumors with stromal invasion between 3.1 and 5.0 mm). The case with recurrence in our study had stromal invasion of 3mm, similar to studies that describe recurrences only in patients with stromal invasion between 1 and 3mm. 9 14 35 The recurrence was observed at 30 months and falls according to other studies that report the risk of recurrence peaking up to 36 months. 11 14 16 31 Some authors reported that subsequent recurrences (many years later) are possibly related to inadequate follow-up (unsatisfactory colposcopies, scar changes, or stenosis) or new lesions (new cancer), not correlated with progression. 9 10 12 16

All patients in the current study had negative margins and an absence of LVSI. According to some studies, the higher recurrence risk occurs in cases with LVSI and positive margins. 9 13 14 31 35 Wong et al. 35 reported positive surgical margins in CKC or LEEP as an independent risk factor for residual disease in early invasive cervical cancer stage. They reviewed the pathology reports of 108 hysterectomy specimens. Only two patients were treated with conservative fertility-sparing surgery and there was no recurrence on follow-up (mean follow-up: 63.5 months). Östör and Rome 9 found recurrence only in cases with positive margins. Kim et al. 13 showed that only cases with endocervical positive margins recurred in their study. Qian et al. 14 included 280 patients with stage IA1 cervical cancer within epidermoid, glandular, and clear cell histological types, regardless of LVSI. They found a recurrence rate of 2.4% in patients treated with conization. 14

The findings from our study concur with the standard management even in a developing country. 4 5 7 8 This practice has been reassured by similar studies comparing conization and hysterectomy outcomes for stage IA1 CC. 14 16 32 Since we had only one recurrence, it is not possible to make a direct correlation with the known risk factors for CC. This study is limited by retrospective data collection and data from a single institution with possible referral bias. Another potential limitation is the absence of a control group, which could add relevance to the data. The strengths of this study include a homogeneous group of patients from a developing country within the perspective of surgical treatment, a strict follow-up routine, and a long period of follow-up.

## Conclusion

This study was conducted in Brazil, a developing country with high rates of cervical cancer, and we found patients with stage IA1 CC without LVSI and with negative margins treated by conservative treatment resulting in excellent outcomes since it was found no recurrences of invasive cancer or any cancer-related deaths.
